# Improving the role of immune checkpoint inhibitors in the management of advanced urothelial carcinoma, where do we stand?

**DOI:** 10.1016/j.tranon.2022.101387

**Published:** 2022-03-09

**Authors:** Hélène Houssiau, Emmanuel Seront

**Affiliations:** Department of Medical Oncology, Centre Hospitalier de Jolimont, Rue Ferrer 159, 7100 Haine Saint Paul, Belgium

**Keywords:** Urothelial carcinoma, Immune checkpoint inhibitors, Maintenance strategy, PD-L1, Platinum-based strategy

## Abstract

Immune checkpoint inhibitors (ICIs) have significantly improved the outcome of patients with metastatic urothelial cancer (mUC). If these agents were first used in monotherapy after failure of platinum-based chemotherapy, new strategies have been evaluated in the last years, including association of ICIs, ICI plus chemotherapy association or maintenance therapy. This maintenance concept allows a better management of mUC, which is particularly interesting in cisplatin-ineligible patients.

This paper aims to review the current knowledge of ICIs in urothelial carcinoma as well as the new indications of these agents in mUC.

## Highlights

• Immune checkpoint inhibitors improve outcome in metastatic urothelial carcinoma.
• Maintenance strategy involves early use of avelumab after efficient chemotherapy.
• Patients with no progression on chemotherapy should receive maintenance avelumab.
• There is no place for PD-L1 testing when considering maintenance strategy.

## Graphical abstract

**Figure:**
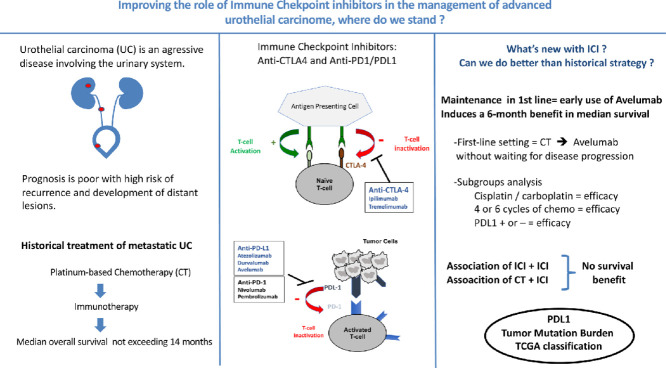

## Introduction

Bladder cancer is the seventh most common cancer with more than 350,000 newly diagnosed cases, and approximately 150,000 deaths each year worldwide [1]. Urothelial carcinoma (UC) histology accounts for more than 90% of bladder cancer; other histologies include squamous carcinoma, adenocarcinoma and neuroendocrine carcinoma [1,2]. Around 75% of patients present with localized and non-muscle invasive tumor (NMIUC) and are treated in a curative intent with different modalities, including surgical resection, intravesical chemotherapy and/or intravesical injection of bacillus Calmette-Guerin (BCG) [3]. Muscle-invasive UC (MIUC) requires multimodal strategy including cystectomy and perioperative chemotherapy [4]. Despite this aggressive management, more than 50% of MIUC patients develop metastases with a poor prognosis.

For decades, cisplatin-based chemotherapy remained the standard treatment in first-line metastatic UC (mUC), improving progression free survival (PFS) and overall survival (OS) of these patients. Two regimens, methotrexate/vinblastine/adriamycin/cisplatin (M-VAC) and cisplatin/gemcitabine (CG), have shown greater activity over cisplatin alone in the first-line setting with an objective response rate (ORR) of 40–49%, a PFS ranging from 7.7 to 10 months and an OS not exceeding 14.8 months [5]. Due to its better tolerability and safety profile, the CG combination remains thus the standard of care for mUC patients in first-line setting. A proportion of patients are ineligible for cisplatin due to poor performance status and renal failure and are treated with carboplatin-gemcitabine regimen, with inferior efficacy, ORR not exceeding 36%, PFS 5 months and OS 9 months [4], [5], [6].

Platinum-based chemotherapy does not cure patients and progression occurs in more than 90% of patients. Second-line options are limited and include mainly immune checkpoint inhibitor (ICI) in monotherapy with only a limited percentage of patients presenting durable benefit [7], [8], [9]. Further therapeutic options are available, including paclitaxel, docetaxel and vinflunine, but these agents do not improve significantly outcome of these patients [10], [11], [12].

Advances in immuno-oncology field has considerably improved outcome of patients in different cancer types, including UC. These treatments allow innovative strategies including maintenance treatment and tailored treatment. We describe in this review the recent advances in mUC management with ICIs.

### Rational for immunotherapy in bladder cancer

Retrospective analysis showed that patients with increased tumor-infiltrating CD8+ lymphocytes (TILs) within advanced UC (pT2, pT3, or pT4) have better disease-free survival and OS than patients with similar-staged UC and fewer intra-tumoral CD8+ TILs, suggesting that this lymphocyte infiltration is associated with better outcome [13,14]. This highlights the role of immune system in controlling evolution of UC cells, which is indirectly confirmed by the fact that intravesical instillations of bacillus Calmette- Guerin (BCG) prevent recurrences of high-risk NMIUC [15].

Immune system is able to detect and eliminate cancer cells, as they exhibit differences in antigenicity from healthy cells. Tumor cells release tumor-associated antigens, named neoantigens that are captured by antigen-presenting cells (APC) through the major histocompatibility complex (MHC) I. APC migrate to lymphoid organs, where they activate effector T-cells, by presenting these neoantigens to the surface T-cell receptor (TCR). Activated T-cell in turn infiltrate tumors, and kill cancer cells. Interestingly, UC carries the third highest mutation rate of all studied cancers, resulting in production of high amount of neoantigens, which is required for antigenicity and effective immune response [16].

The immune synapsis established by the TCR with MHC is regulated by range of positive (co-stimulatory signals) and negative (co-inhibitory signals) interactions between the T-cell and the antigen presenting cell. The main positive regulatory interaction is provided by the binding of B7 on the surface of APC to CD28 on T-cells, leading to the recruitment and activation of Src/like tyrosine kinases that, in turn, activates the phospho-inositol-3 phosphate kinase (PI3K)-Akt axis and the MAPK-ERK and Janus Kinase (JNK) signaling pathway. All these pathway enhance activation of inflamatory reaction through NF-kβ activation and Il-2 production [17,18].

However, malignant cells develop different mechanisms to evade immune recognition; one such strategy involves the expression of cell-surface molecules, named immune checkpoints, on tumor and tumor-specific lymphocytes, that are able to inhibit activated T-cells. The most commonly investigated immune checkpoints are cytotoxic T lymphocyte-associated protein 4 (CTLA-4), programmed cell death protein-1 (PD-1) and programmed death ligand-1 (PD-L1). CTLA-4 expressed on T-cell exerts its inhibitory effect by competing with CD28 and by binding to B7, resulting in T-cell inactivation in lymphoid tissues.

**Fig. 1:**
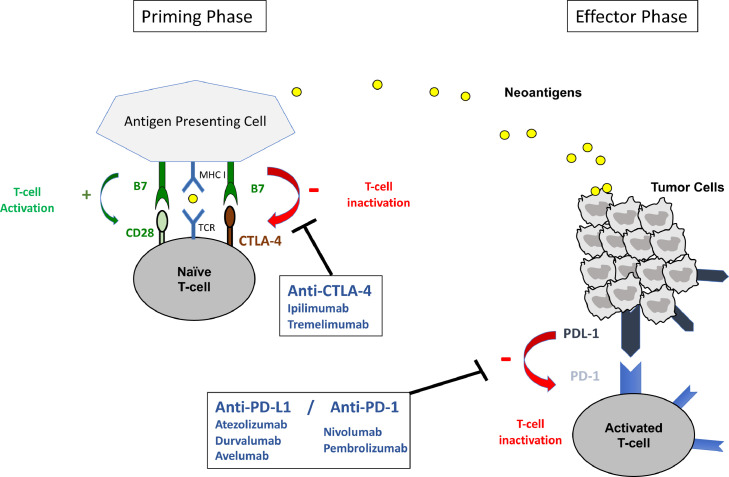
Neoantigens, released by tumor cells are captured by antigen-presenting cells (APC) through the major histocompatibility complex (MHC) I. APC migrate to lymphoid organs, where they activate effector T-cells, by presenting these neoantigens to the surface T-cell receptor (TCR). Activated T-cell in turn infiltrate tumors, and kill cancer cells by enhancing inflammatory reaction. The main positive regulatory interaction that activate T-cells is provided by the binding of B7 on the surface of APC to CD28 on T-cells.

In a same way, PD-1 is an inhibitory receptor expressed on T-cells. PD1 can suppress T cell activation by affecting T cell proliferation. PD1 relieves inhibition of PTEN, a phosphatase that inhibits the phosphorylation of phosphatidylinositol [3], [4], [5] triphosphate (PIP3) and thus prevents the activation of AKT by PI3K. Furthermore, PD1 can inhibit cyclin-dependent kinase (CDKs), resulting in the arrest of T cell proliferation [17,18]. PD1 can also control negatively the surface expression levels of the TCR, avoiding the recognition of pMHC by the T cell. Binding of PD1 to PD-L1 induces activation of these pathways and inactivation of tumor-specific T cells. Upregulation of PD-L1 by cancer cells can be enhanced by excessive activation of oncogenic pathways (PI3K-Akt axis or MAPK cascade) and/or loss of tumor supressor genes (*PTEN*) and could be mediated by inflammatory cytokines such as interferon *γ* [19,20]. (Fig. 1)

The expression of PD1 and PD-L1 by UC cells and infiltrating immune cells is associated with poorer outcomes. Levels of PD-L1 expression in NMIUC have been correlated with bladder cancer higher-stage, higher frequencies of postoperative recurrence and poorer survival [21].

Immune checkpoint inhibitors (ICIs) are monoclonal antibodies that target immune checkpoints, and thereby disrupt the inhibitory signals and reactivate immune system. The binding of antibodies and antigens is very specific, but immune checkpoint inhibitors are antibodies that differ in their specificity.

Two monoclonal antibodies targeting CTLA-4 have been developed: ipilimumab and tremelimumab. Atezolizumab, durvalumab and avelumab are three anti-PD-L1 inhibitors; they were developed with IgG1 isotype. Compared to durvalumab and atezolizumab, avelumab can, in addition to PD-L1 inhibition, induce antibody-dependent cell mediated cytotoxicity (ADCC), which results in a direct lysis of tumor cells [22]. The two anti-PD1 inhibitors, pembrolizumab and nivolumab, belong to IgG4 subclass of human antibodies. IgG4 subtypes have a much lower potency to mediate ADCC than other IgG subtypes.

Improvement in the management has been done in mUC, including the first-line management. This review offers a new perspective on the mUC management.

### What is the current standard of care in first line mUC ? The concept of maintenance strategy

The maintenance strategy, meaning starting ICI directly after first-line chemotherapy without waiting for disease progression, is the current standard of care in 2021, approved by the Food and Drug Administration (FDA) and European medical Agency (EMA). This approval was based on the results of the randomized Phase III JAVELIN Bladder 100 trial. This study enrolled 700 patients with unresectable locally advanced or mUC who had not experienced disease progression after a first line platinum-based chemotherapy. Enrolled patients had to present complete response (CR), partial response (PR) or stable disease (SD) after 4–6 cycles of cisplatin or carboplatin plus gemcitabine. Patients were randomized (1:1) to receive maintenance treatment with avelumab (10 mg/kg IV q2w) and best supportive care (BSC) or only BSC. The primary end point was OS, which was assessed in both the overall population and the PD-L1–positive population. Fifty-one patients of patients were PD-L1 positive (PD-L1 was considered as positive if at least one of the following three criteria were met: at least 25% of tumor cells stained for PD-L1, at least 25% of immune cells (ICs) stained for PD-L1 if more than 1% of the tumor area contained ICs, or 100% of ICs stained for PD-L1 if no more than 1% of the tumor area contained ICs) (see Table 1 for PD-L1 positivity).

**Table 1:** Criteria for PD-L1 positivity across different trials.

Study design	Target	Agent	Definition of PD-L1 positivity
Phase III JAVELIN Bladder 100 [23]	PD-L1	Avelumab	≥ 25% of TC OR ≥ 25% of ICs if >1% of tumor area contained ICs OR 100% of ICs if ≤1% of Tumor area contained ICs
Phase III IMvigor 130 [26]Phase II IMvigor210(Cohort1 and 2) [[30], [31], [32],^37^]Phase III IMvigor 211 [38]	PD-L1	Atezolizumab	≥ 5% of tmor infiltrating ICs in tumor area
Phase III KEYNOTE 361 [27]Phase III KEYNOTE-045 [40]Phase II KEYNOTE-052 [33,34]	PD1	Pembrolizumab	CPS ≥10 (CPS =% of PD-L1 + IC and TC related to numbers of tumor cells)
Phase III DANUBE [28]	PD-L1	Durvalumab	≥25% of TC with membrane staining Or ≥ 25% of ICs at any intensity if >1% of tumor area contained ICs OR 100% of ICs at any intensity if 1% of tumor area contained ICs
Phase I-II CheckMate032 [35]	PD1 and CTLA-4	Nivolumab + Ipilimumab	≥1% or ≥5% of TC in a section that included ≥100 evaluable TC
Phase II Checkmate 275 [39]	PD1	Nivolumab	≥1% or ≥5% TC

TC=tumor cells, ICs=immune cells, TA=tumor area, PD1=programmed cell death 1, PD-L1=programmed cell death ligand 1; CPS = combined positive score.

Overall survival was significantly improved with avelumab + BSC compared to BSC alone in the overall population (21.4 vs 14.3 months; Hazard Ratio [HR], 0.69; 95% confidence interval [CI] 0.56–0.86; *P* = 0.0005). The 12-month OS was 71.3% in the avelumab + BSC group compared to 58.5% in the BSC group. In PD-L1-positive patients, OS was also significantly improved in the avelumab + BSC group compared to BSC group (Not Reached (NR) vs 17.1 months, HR 0.56, 95% CI 0.40–0.79; *P* = 0.0003). The 12-month OS was 79.1% in the avelumab group compared to 60.4% in the control group. The PFS was increased in the overall population with the maintenance avelumab strategy compared to control group (3.7 vs 2 months; HR 0.62, 95% CI 0.52–0.75) and in the PD-L1 positive patients (5.7 vs 2.1 months; HR 0.56, 95% CI 0.43–0.73). Response rate was increased in the avelumab + BSC group compared to BSC group, in term of CR (6% vs 0.9%, respectively) and PR (3.7% vs 0.6%, respectively). This increased response rate with avelumab + BSC compared to BSC group was also observed in PDL-1-positive patients (CR 9.5% vs 0.6% and PR 4.2% vs 0.6%, respectively). Importantly, 43.7% of patients randomized to the BSC arm were further treated with ICIs, highlighting the fact that introducing avelumab before progression of the disease, directly after chemotherapy, improves survival compared to waiting for disease progression after chemotherapy [23] Table 2. summarizes results of different trails presented in this review.

**Table 2:** Pivotal trials that evaluated immune checkpoint inhibitors in advanced urothelial carcinoma.

	Setting	Agent	N	ORR	PFS (months)	OS (months)
Phase III randomized JAVELIN Bladder 100 [23]	Maintenance setting after response or stable disease on first-line platinum-based therapy	Avelumab	350	All pts = 9.7%, CR = 6%High PD-L1 =13.8%, CR =9.5%	All pts =3.7 High PD-L1= 5.7	All pts = 21.4; 1-y OS =71.3%High PD-L1 = NR; 1-y OS =79.1%
		BSC	350	All pts = 1.4%, CR=0.9%High PD-L1=1.2%, CR=0.6%	All pts = 2High PD-L1 =2.1	All pts = 14.3; 1-y OS =58.5%High PD-L1=17.1; 1-y OS =60.4%
Phase IIIRandomizedIMvigor 130 [26]	1st-line mUC	*Atezolizumab + plt/gem*	451	All pts = 48%, CR=13%	All pts = 8.2 High PD-L1 = 8.6	All pts = 16
		Atezolizumab	362	All pts = 23%, CR 6%	NA	All pts = 15.7 High PD-L1= 27.5
		*Placebo + plt/gem*	400	All pts = 44%, CR =7%	All pts = 6.3 High PD-L1 = 6.3	All pts = 13.4
Phase IIIRandomizedKEYNOTE- 361 [27]	1st-line mUC	*Pembrolizumab + plt/gem*	351	All pts = 54.7%, CR =15%,High PD-L1 = 57.2%, CR =16%	All pts = 8.3High PD-L1 = 8.6	All pts = 171-y OS =62%
		Pembrolizumab	307	All pts = 30.3%, CR = 11% High PD-L1 = 32.5%, CR = 13%	NA	All pts = 15.6High PD-L1 = 16.1
		*Plt/gem*	352	All pts = 44.9%, CR =12% High PD-L1 = 46.2%, CR= 17%	All pts = 7.1 High PD-L1 = 8.6	All pts = 14.31-y OS =56%
Phase IIIRandomizedDANUBE [28]	1st-line mUC	Durvalumab + Tremelimumab	342	All pts = 36%, CR = 8%High PD-L1 = 47%, CR = 12%	All pts = 3.7High PD-L1 = 2.4	All pts = 15.1High PD-L1 = 17.9
		Durvalumab	346	All pts = 26%, CR =8%High PD-L1 = 28%, CR =10%	All pts =2.3High PD-L1 =2.4	All pts =13.2High PD-L1 =14.4
		*plt/gem*	344	All pts = 49%, CR = 6%High PD-L1 = 48%, CR = 7%,	All pts = 6.7High PD-L1 = 5.8	All pts = 12.1High PD-L1 = 12.1
Phase IISingle-agentIMvigor 210Cohort 1 [30], [31], [32]	1st-line mUC (cisplatin ineligible)	Atezolizumab	119	All pts = 24%, CR = 7%High PD-L1 = 24%	All pts = 2.7High PD-L1 = 4.1	All pts = 15.9High PD-L1= 12.3Low PD-L1= 19.1
Phase IISingle-agentKEYNOTE −052 [33,34]	1st-line mUC (cisplatin ineligible)	Pembrolizumab	370	All pts= 29%, CR = 7%	All pts = 26-mth PFS = 30%	All pts = 11.3High PD-L1 = 18.5
Phase I-II CheckMate 032 [35]	After failure of platinum-based therapy	N1/I3	61	All pts = 23%, CR = 2%	All pts = 4.3	All pts = 10.2
		N3/I1	54	All pts = 19%, CR = 0%	All pts = 2.6	All pts = 7.3
Phase IISingle-agentIMvigor 210Cohort 2 [37]	After failure of platinum-based therapy	Atezolizumab	310	All pts = 16%, CR = 7%High PD-L1 = 28%, CR = 15%	All pts = 2.1	All pts = 7.9; 1-y OS = 37%High PD-L1= 11.9; 1-y OS =50%
Phase IIIRandomizedIMvigor 211 [38]	After failure of platinum-based therapy	Atezolizumab	467	All pts = 14%, CR = 4%High PD-L1 = 23%	All pts = 2.1High PD-L1 = 2.4	All pts = 8.9; 1-y OS = 40%High PD-L1 = 11.1
		Vinflunine or paclitaxel or docetaxel	464	All pts = 15%, CR = 4%High PD-L1 = 22%	All pts = 4High PD-L1 = 4.2	All pts = 8.2; 1-y OS = 33%High PD-L1 = 10.6
Phase IISingle-agentCheckmate 275 [39]	After failure of platinum-based therapy	Nivolumab	265	All pts =19.6%High PD-L1 = 28.4%Low PD-L1 = 16.1%	All pts = 2	All pts = 8.7; 1-y OS = 41%High PD-L1 = 11.3; Low PD-L1 =5.9
Phase IIIRandomizedKEYNOTE −045 [40]	After failure of platinum-based therapy	Pembrolizumab	270	All pts = 21.1%, CR = 7%	All pts = 2.1	All pts = 10.1High PD-L1 = 8
		Vinflunine or Paclitaxel or Docetaxel	272	All pts = 11.4%, CR = 3.3%	All pts = 3.3	All pts = 7.4High PD-L1 = 5.2
Phase I/IISingle agent [42]	After failure of platinum-based therapy	Durvalumab	182	All pts = 17%High PD-L1 = 26.3%Low PD-L1 = 4.1%	NA	All pts = 14.1; 1-y OS = 50%
Phase IbSingle agent(Javelin) [43,44]	After failure of platinum-based therapy	Avelumab	242	All pts =16.1%High PD-L1 = 25%Low PD-L1 = 14.7%	NA	All patients = 7.4; 1-y OS = 54.9%

PFS=progression free survival; ORR=objective response rate; CR=complete response; OS=overall survival; pts=patients; *y*= year; mth = month; BCS=best supportive care; NR=not reached; ICI=immune chekpoint inhibitor; PR=partial response; ptl =platinum agent (cisplatin or carboplatin); *gem*=gemcitabin; NE=not estimable; N1/I3=nivolumab 1 mg/kg + ipilumimab 3 mg/kg; N3/I1=nivolumab 3 mg/kg + ipilimumab 1 mg/kg ; N3=nivolumab 3 mg/kg ; NA = not available.

Post-hoc analyses showed OS benefit irrespective of duration/cycles of chemotherapy (4 to 6 cycles). To date, no evidence is available to demonstrate the efficacy of avelumab maintenance after more or fewer cycles of first-line platinum-based chemotherapy, and data from prospective or real-world studies are needed to identify optimal strategy. Furthermore, the amplitude of response to first-line chemotherapy did not influence the response to avelumab maintenance; all patients were stratified according to achievement of objective response (CR or PR) or SD with chemotherapy, and the OS benefit was similar in these subgroups (HR 0.69, 95% CI 0.53–0.89) and 0.70 (95% CI 0.46–1.05), respectively. All other subgroups benefited from avelumab maintenance; prolonged OS was observed in the avelumab + BSC group compared to BSC group in patients with upper or lower tract tumors, metastatic or locally advanced and unresectable disease (prior to chemotherapy), and lymph node-only disease.

No evidence is available to support whether a maximum or fixed duration of avelumab treatment would be beneficial. In the JAVELIN Bladder 100 trial, the median duration of avelumab treatment at data cut-off (October 2019) was 24.9 weeks (range from 2.0 to 159.9 weeks). The tolerability of avelumab was supported by patient-reported outcome (PROs) data that were similar in patients in the avelumab plus BSC group and in the BSC group. Furthermore, median time to deterioration in the Functional Assessme1nt of Cancer Therapy Bladder Cancer Symptom Index-18 (NFBISI-18) disease-related symptoms–physical subscale was similar between arms [24,25].

There was no new safety signal with avelumab maintenance and adverse events (AEs) profile was similar to that observed in ICI trials in mUC. AEs of any grade occurred in 98.0% in the avelumab group and in 77.7% in the control group, with adverse events of grade ≥3 occurring in 47.4% and 25.2%, respectively. In the avelumab group, adverse events led to treatment discontinuation in 11.9%. Death was attributed by the investigator to the toxicity of trial treatment in two patients (0.6%) in the avelumab group. Amongst the avelumab-treated patients, 29.4% had an immune-related adverse event including 7.0% with a grade 3 event. The most frequent category of immune-related AE was thyroid disorders. High-dose glucocorticoids (≥40 mg total daily dose of prednisone or equivalent) were administered after an immune-related adverse event in 9.0% who received avelumab [23].

### Evaluation of other strategies in first-line mUC: is there a role in combining chemotherapy and ICI?

Two trials evaluated the benefit to combine ICI and chemotherapy in mUC. These two trials used a similar design and, in the same time, evaluated the efficacy of ICI monotherapy.

The IMvigor130 trial is a multicentric phase III trial that randomized patients with locally advanced or mUC in 3 groups: atezolizumab (1200 mg IV q3w) plus chemotherapy (cisplatin/carboplatin plus gemcitabine; group A); atezolizumab (1200 mg IV q3w alone; group B) and placebo plus chemotherapy regimen (group C). The choice between carboplatin and cisplatin, as well as the number of cycles, was left to the treating team. Atezolizumab was pursued until progression (according to RECIST criteria) or unacceptable toxicity. The co-primary endpoints were PFS and OS between groups A and C and OS between groups B and C in a hierarchical approach, meaning that this last endpoint was statistically evaluated only if the OS of group A was statistically superior to group C. When focusing on the benefit of combining ICI plus chemotherapy in the intention-to-treat (ITT) population, PFS tended to be increased in group A compared to group C (8.2 vs 6.3 months, respectively; *P* = 0.007). Response was observed in 47% of patients in group A and in 44% of patients in group C, including CR in 13% and 7%, respectively. In patients who had a response, the median duration of response (DOR) was 8.5 months (95% CI 7.2–10.4) in group A and 7.6 months (95% CI 6.3–8.5) in group C. The median OS did not statistically differ between group A and C, with a median survival of 16 months and 13.4 months respectively (HR 0.83, 95% CI 0.69–1.00; *P* = 0.027). Subgroup analyses showed that patients treated with cisplatin, with higher expression of PD-L1 and higher ECOG performance status experienced a greater benefit [26].

The KEYNOTE 361 trial is a randomized, open-label, phase III trial that randomized around 1000 patients with untreated locally advanced and unresectable or mUC in three arms: pembrolizumab plus chemotherapy (6 cycles of cisplatin/carboplatin plus gemcitabine; group A), pembrolizumab monotherapy (200 mg IV q3w; Group B), and chemotherapy (6 cycles of cisplatin/carboplatin plus gemcitabine; Group C). Pembrolizumab was given for 35 cycles as monotherapy and for 29 cycles in the combined treatment arm as maintenance after 6 courses of chemotherapy. The co-primary endpoints were PFS and OS between group A and C for the total patient population, and, in a hierarchical approach, OS between group B and C in patients with Combine Positive Score (CPS) ≥10 and in the total population; this last endpoint was tested only if the PFS and OS was superior in group A compare to group C. CPS was measured as the percentage of PD-L1–positive immune and tumor cells compared to the number of tumor cells (see Table 1 for PD-L1 positivity. The addition of pembrolizumab to first-line platinum-based chemotherapy did not significantly improve PFS in the total population per the prespecified *P*-value boundary of 0.0019; median PFS was 8.3 months in Group A versus 7.1 months in group C (HR 0.78, 95% CI 0.65–0.93; *P* = 0•0033). At 6, 12, 18, and 24 months, the estimated proportion of patients who were alive and progression-free was 74%, 34%, 23% and 20% respectively in the group A, and 70%, 21%, 14% and 14% respectively in the group C. The addition of pembrolizumab to first-line platinum-based chemotherapy did not significantly improve OS in the total population per the prespecified p-value boundary of 0.014; median OS was 17.0 months in group A versus 14.3 months in group C (HR 0.86, 95% CI 0.72–1.02, *P* = 0•0407). At 12 months, the estimated proportion of patients who were alive was 62% in group A and 56% in group C. The response rate reached 54.7% in group A and 44.9% in group C, including CR rate of 15% and 12%, respectively. Response rate was more important with cisplatin compared to carboplatin in group A (64.1% vs 47.2%) and in group C (48.7% vs 41.8%). The median DOR was 8.5 months (8.2–11.4) in group A and 6.2 months (5.8–6.5) in group C. Due to the lack of significance, no further formal statistical hypothesis testing was performed [27] (see Table 1 for PD-L1 positivity).

These two studies did not allow to conclude that adding ICI to chemotherapy in first-line metastatic setting is beneficial compared to chemotherapy alone in patients with untreated, locally advanced, unresectable or mUC.

It is interesting to note that, compared to JAVELIN bladder 100 trial, ICIs were also given in maintenance in IMvigor 130 and KEYNOTE 361 trials but did not result in improve OS compared to chemotherapy alone. This could be explained by the fact that in JAVELIN 100 trial, patients with PD after chemotherapy were excluded, highlighting selection of patients with better prognosis. Concomitant administration of chemotherapy could also be associated with potential immunosuppressive effect that could decrease efficacy of ICI in UC; conversely, in the JAVELIN trial, avelumab was started 4–10 weeks after the last chemotherapy cycle, which could appear as the optimal time to start maintenance after chemotherapy. Finally, the proportion of patients receiving cisplatin-based chemotherapy, as opposed to carboplatin, was higher in the JAVELIN trial, when compared to both IMvigor 130 and KEYNOTE 361, which may also have played a role in the discrepant survival results.

Association of chemotherapy and ICI did not result in increased rate of toxicity. In the IMvigor 130 trial, grade 3 and 4 AEs were observed in 81% of patients treated with the association and in 81% of patients treated with chemotherapy alone. In KEYNOTE 361, grade 3 and 4 AEs were observed in 87% in the association group and in 82% of patients in the chemotherapy group [26,27].

### Evaluation of other strategies in first-line mUC: is there a place for ICI monotherapy in mUC patients?

Three randomized trials evaluated the role of ICI monotherapy in first-line setting. IMvigor 130 and KEYNOTE-361 trial compared to atezolizumab and pembrolizumab alone, respectively to chemotherapy. In both trials, the association of ICI plus chemotherapy appeared not statistically superior to chemotherapy in term of PFS and OS. As these endpoints were not met, the hierarchical approach did not allow to statistically evaluate the difference between ICIs monotherapy and chemotherapy alone in these trials and the results were purely exploratory.

In IMvigor 130 trial, there was a numerical increase in the median OS for atezolizumab monotherapy compared with placebo plus chemotherapy (15.7 vs. 13.1 months; HR 1.02, 95% CI 0.83–1.24), but this benefit emerged late and in the first months of treatment, chemotherapy seemed to be more efficient in delaying death. This could be explained by the lower ORR in atezolizumab compared to CT (23.4% vs 44.1%, respectively). However, the median DOR was more than 3.5 times longer for atezolizumab monotherapy than for chemotherapy (29.6 vs 8.1 months, respectively). This study therefore did not allow to conclude that atezolizumab given as monotherapy and in first-intent is efficacious in all-comers patients with locally advanced or mUC. However, atezolizumab appeared more beneficial in PDL1-positive patients, but no formal statistical comparison could be done; exploratory subgroups analyses demonstrated that the median OS for the PD-L1 IC2/3 (defined as PD-L1 expression on ICs ≥5% of IC) patients was higher in the atezolizumab monotherapy arm than in the placebo-chemotherapy arm (27.5 vs 16.7 months, respectively). Atezolizumab monotherapy was better tolerated than chemotherapy alone, with grade 3–4 AEs occurring in 15.3% versus 80.8% of patients, respectively and with AEs leading to treatment discontinuation occurring in 6.2% versus 33.8% of patients [26].

In KEYNOTE-361 trial, when analyzing the total population, irrespective of CPS, the median OS was 15.6 months (95% CI 12.1–17.9) in the pembrolizumab group versus 14.3 months (95% CI 12.3–16,7) in chemotherapy group (HR 0.92, 95% CI 0.77–1.11). As observed in the IMvigor 130 study, efficacy of pembrolizumab appeared late and in the first months, chemotherapy appeared superior to pembrolizumab; this could be due to lower response rate with pembrolizumab alone compared to chemotherapy (30.3% vs 44.9%, respectively). However, the median DOR was longer with pembrolizumab compared to chemotherapy (28.2 months vs 6.2 months, respectively). Pembrolizumab benefit in term of OS was not superior to chemotherapy in patients with CPS ≥10; the median OS reached 16.1 months in the pembrolizumab group compared to 15.2 months in the chemotherapy group. This trial did not show superiority of pembrolizumab monotherapy compared to platinum-based therapy in first-line metastatic setting both in all-comers and in PDL1-positive patients [27].

A third trial, the randomized phase III trial DANUBE, evaluated in a three arms fashion the benefit of durvalumab (1500 mg q4w) monotherapy, durvalumab plus tremelimumab versus chemotherapy. The co-primary endpoints were OS compared between durvalumab monotherapy and chemotherapy alone in PD-l-positive patients (≥25% of tumor cells or immune cells) and OS compared between the durvalumab plus tremelimumab and chemotherapy alone in the ITT population. The median OS in the durvalumab group was lower than in the chemotherapy group in the PD-L1-positive population (14.4 vs 12.1 months, respectively; HR: 0.89, 95% CI 0.71–1.11; *P* = 0.30). In a similar way than with atezolizumab and pembrolizumab monotherapy, durvalumab efficacy appeared late compared to chemotherapy, due to a lower response rate with durvalumab compared to chemotherapy (26% vs 46%, respectively). The secondary endpoint median PFS in the ITT population was 2.3 months in the durvalumab group compared to 6.7 months in the chemotherapy group. In the PD-L1-positive population, median PFS was 2.4 months and 5.8 months, respectively (see Table 1 for PD-L1 positivity) [28].

Even if majority of these results focusing on efficacy of ICI monotherapy are exploratory, these different trials seem to conclude in a poor efficacy of ICI monotherapy compared to chemotherapy in all-comers patients with mUC. Another important point is that response rate is more important with chemotherapy compared to ICI monotherapy. This highlights the fact that platinum-based chemotherapy remains, in patients eligible for platinum, the standard of care in this setting.

### Management of cisplatin-ineligible patients in mUC

Significant proportion of patients, both in perioperative or metastatic setting, are ineligible to cisplatin due to renal insufficiency and/or poor performance status. In these patients, carboplatin-based regimen remained the standard of care in first-line metastatic setting for a long time. However, carboplatin-based chemotherapy has been demonstrated to be inferior compared to cisplatin-based regimen in term of response rate, PFS and OS [29]. However, due to limited option, no other option is available for these patients.

Two phase II trials were dedicated to evaluate ICI monotherapy in cisplatin-ineligible patients. The cohort 1 of the phase II IMvigor210 study enrolled 119 who were ineligible for cisplatin-based chemotherapy and who had received no prior chemotherapy in metastatic setting. Patients were deemed cisplatin-ineligible by at least one of the following criteria: Glomerular filtration rate > 30 and < 60 mL/min, ≥ G2 hearing loss or peripheral neuropathy or ECOG performance status (PS) 2. Seventy percents were cisplatin ineligible due to renal impairment, 56% and 15% had 1and 2 Bajorin risk factor (poor performance status and visceral metastases), respectively. PD-L1 scoring criteria designated tumors as IC0, IC1, or IC2/3 (PD-L1 expression on <1%; ≥1% and <5%; or ≥5% of IC, respectively) (see Table 1 for PD-L1 positivity); 32% of patients had high PD-L1 expression (IC2/3). After a median follow-up of 70.8 month, the ORR was 23.5% in all patients, including 8% of CR. The response rate by PD-L1 subgroup reached 28.1% in IC2/3 and 21.8% in IC0/1. Median time to onset of first response was 2.1 months (1.8–10.5 months). Median response duration had not been reached in all patients or in pre-defined PD-L1 subgroups (range 3.7 to 21.0). Fifty-four percents of the responders in the total cohort had an ongoing response at the time of the current analysis, as did 47.4% and 66.7% of the responders in the PD-L1 low and high subgroups, respectively; the corresponding median DOR were 59.1 months, 53.5 months, and unreached, respectively. The median PFS was 2.7 months (95% CI 2.1–4.2) in all patients, 4.1 months (95% CI 2.3–11.8) in IC2/3 patients, 2.1 months (95% CI 2.1–5.4) in IC1 patients, and 2.6 months (95% CI 2.1–5.7) in IC0 patients. The median OS was 16.3 months (95% CI 10.4 to not estimable) in all patients, 12.3 months (95% CI 6.0 to not estimable) in IC2/3 patients, and 19.1 months (95% CI 9.8 to not estimable) in IC0/1 patients. The 12- and 60-month survival rate were 57% and 21.6% in all patients, respectively. Bajorin risk factors also appeared as a prognostic tool in ICI era. Median survival was not reached in patients with no risk factors, was 13.4 months in those with 1 risk factor (either visceral metastases or ECOG PS 2), and was 6.2 months in those with 2 risk factors [30], [31], [32].

The phase II trial KEYNOYTE-052 evaluated pembrolizumab as first-line agent in 370 ciplatin-ineligible patients (28.9% were ≥ 80 years, 41.9% of patients with ECOG PS 2, 49% with renal dysfunction, 85.1% with visceral disease, 35% with ECOG PS 2 and visceral metastatic disease and 10% with ECOG PS 2 and renal dysfunction). These patients received pembrolizumab 200 mg intravenously every 3 weeks for up to 24 months. The median time from enrollment to data cut-off was 11.4 months (range, 0.1–41.2 months). The primary endpoint ORR reached 28.6%, including 8.9% CR and 19.7% PR. The disease control rate, combining CR, PR and SD reached 46.8%. The median time to response was 2.1 months (1.3–9.0 months) and the median DOR was 30.1 months (95% CI 18.1 months -NR); responses lasted ≥ 12 and ≥ 24 months in 67% and 52% of patients, respectively. The median PFS was 2 months (95% CI 2.0–3.0), with a 6-month PFS of 30%. The median OS was 11.3 months (95% CI, 9.7–13.1), with a 12- and 24-month OS rate of 46.9% and 31.2%, respectively. In patients with CPS ≥ 10, ORR was 47.3% and median OS was 18.5 months (95% CI, 12.2–28.5 months). CPS positivity was associated with better outcome; in PD-L1 CPS ≥ 10 patients, ORR was 47.3% (CR 20.0% and PR 27.3%) compared to 20.3% in PD-L1 CPS ≤10. The median DOR for the CPS ≥ 10 and CPS < 10 subgroups was NR (95% CI, 18.1 months -NR) and 18.2 months (95% CI, 9.7 months -NR), respectively. In the CPS ≥ 10 and CPS < 10 subgroups, median OS was 18.5 months (95% CI, 12.2–28.5 months) and 9.7 months (95% CI, 7.6–11.5 months), respectively; 24-month OS rates were 47.0% and 24.0%, respectively. Objective responses were noted both in patients ECOG PS 0/1 (30.4%) and in patients with ECOG PS 2 (26.3%). Median OS for ECOG PS 0/1 and ECOG PS 2 was 13.0 months (95% CI 11.0–16.5 months) and 9.6 months (95% CI 5.7–11.5 months), respectively. In patients with ECOG PS 2 and visceral metastatic disease, ORR was 22.3%, median OS was 7.8 months (95% CI, 5.1–10.6 months), and median DOR was 24.0 months (95% CI, 7.5 months -NR) [33,34].

Despite absence of head-to-had comparison with carboplatin-based regimen in cisplatin-ineligible patients, atezolizumab and pembrolizumab monotherapy seem to result in high rate of response and improvement in OS. This led to the approval of atezolizumab and pembrolizumab for cisplatin-ineligible patients in first-line metastatic setting in 2017. However, the data observed in IMvigor 130 trial showing superior benefit of atezolizumab monotherapy in PDL1 positivity led to reserve ICI monotherapy in first-line metastatic setting only in cisplatin ineligible patients with a high PDL-1 expression.

**Fig. 2:**
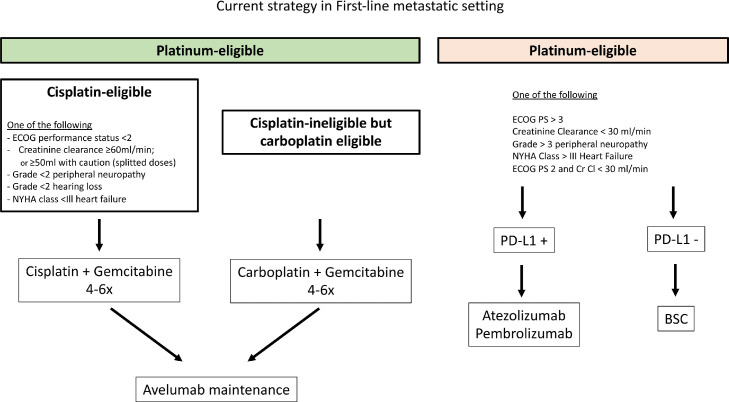
Therapeutic algorithm in advanced urothelial carcinoma management.

With the results of JAVELIN Bladder 100, mUC patient deemed cisplatin-ineligible should receive carboplatin-based regimen (4 to 6 cycles) and then avelumab in maintenance. The subgroup analysis showed benefit both in patients receiving cisplatin or carboplatin-based regimen. The HR for patients receiving avelumab maintenance compared to BSC after cisplatin- and after carboplatin-based regimen was 0.69 (0.51–0.94) and 0.66 (0.47–0.91), respectively. This suggests that, based on the cisplatin eligibility criteria, patients should be treated either with cisplatin or with carboplatin, followed by avelumab maintenance [23] Fig. 2. summarizes the therapeutic management of mUC.

### Evaluation of other strategies in first-line mUC: is there a place for ICIs association ?

Combination of ICIs could appear promising, particularly the combination of PD-L1/ PD-1 inhibitors and CTLA-4 inhibitors as their actions are complementary. CTLA-4 is expressed by regulatory memory CD4+ and T-cells and is functional during the priming phase (early activation of T cells in lymphatic tissues). The PD-1/PD-L1 interaction occurs primarily in peripheral tissues upon representation of antigens to memory T-cells (effector phase). Tremelimumab is a CTLA-4 inhibitor and durvalumab an anti-PDL1 inhibitor. Association of these 2 ICIs has been evaluated in the DANUBE Trial, a multicentre Phase III trial that randomized patients with unresectable, locally advanced or mUC in three groups: durvalumab in monotherapy (1500 mg q4w), durvalumab (1500 mg q4w) plus tremelimumab (75 mg q4w for up to 4 doses followed by durvalumab only as maintenance, and 6-cycles of platinum-based chemotherapy regimen (gemcitabine combined to carboplatin/cisplatin). The co-primary endpoints were OS compared between durvalumab plus tremelimumab and chemotherapy in the ITT population and between durvalumab monotherapy and chemotherapy in PD-L1-positive patients (PD-L1 expression defined as ≥25% of tumor cells or immune cells) (see Table 1 for PD-L1 positivity).

In the ITT population, median OS was 15.1 months in durvalumab plus tremelimumab population and 12.1 months in the chemotherapy group (HR 0.85; 95% CI 0.71–1.02; *P* = 0.075). Chemotherapy resulted in higher ORR compared to durvalumab plus tremelimumab (46% vs 36%, respectively). Interestingly, in the PD-L1 high subgroup (exploratory analysis), ORR was comparable between CT (48%) and durvalumab plus tremelimumab (47%).

Even if the primary endpoint was not met, an exploratory analysis showed that, in PD-L1-positive patients, durvalumab plus tremelimumab resulted in a longer OS compared to chemotherapy (17.9 vs 12.1 months; HR 0.74; 95% CI 0.59–0.93), suggesting that some subgroups of patients could benefit from this ICI combination. Further researches focusing on biomarker are ongoing. The secondary endpoint median PFS, in the ITT population, was shorter in the durvalumab plus tremelimumab group than in the chemotherapy group (3.7 vs 6.7 months). In the PD-L1-positive population, median PFS was 2.4 months and 5.8 months, respectively. Grade 3 or 4 treatment-related AEs were observed in 27% and 60% of the patients treated with durvalumab and tremelimumab combination and chemotherapy, respectively [28].

These results suggests that, today, without any efficient biomarker, ICIs association is not superior to chemotherapy in this head-to-head phase III trial.

Other regimen and dosage schedule have been tested in early clinical trial. As part of the CheckMate 032 trial, the combination of nivolumab plus ipilimumab was evaluated in pretreated patients with locally advanced or mUC who had progressed on ≥1 prior lines of chemotherapy. Patients were treated with nivolumab 1 mg/kg + ipilimumab 3 mg/kg (N1/I3) or nivolumab 3 mg/kg + ipilimumab 1 mg/kg (N3/I1) every 3 weeks for four cycles, followed by nivolumab 3 mg/kg every 2 weeks, or with nivolumab monotherapy 3 mg/kg (N3) every 2 weeks. A higher response rate was observed with N1/I3 (38.5%) compared to other cohorts (26% for N3/I1 and 25.6% for N3). Median DOR has not been reached in any treatment group. The median PFS in the N1/I3 group and in the N3/I1 was 4.3 months and 2.6 months, respectively and the median OS was 10.2 months and 7.3 months, respectively. The rates of grade 3/4 AEs were similar in each group, at 30.8% and 31.7%, for the N1/I3 and N3/N1 arms, respectively [35].

A single-arm phase 2 trial (NCT01524991) evaluated the association of gemcitabine, cisplatin, plus ipilimumab in chemotherapy-naïve patients with mUC. The primary endpoint was the 1-year OS. Thirty-six patients underwent two cycles of cisplatin-gemcitabine alone followed by four cycles of gemcitabine, cisplatin, and ipilimumab. The ORR was 69% with a median OS of 14.6 months and a 1-year OS was 61%, which appeared not superior to historical results of cisplatin-gemcitabine alone. This study thus did not meet the primary endpoint. Grade ≥3 AEs occurred in 81% of patients, the majority of which were hematologic. In a translational analysis that included plasma collection for immunophenotyping, the addition of ipilimumab increased the proportion of CD4+ and CD8+ T*-*cells without depleting T-regulatory or myeloid-derived suppressor cells, suggesting the feasibility of this combination [36].

### Immune checkpoint inhibitiors as second-line agent in mUC

As in many cancer types, ICIs were first evaluated and approved in late stage of the disease; for mUC, they were fist approved in second-line setting, after failure of platinum-based therapy.

Pembrolizumab is the only agent that was shown to be superior to chemotherapy in mUC, based on the KEYNOTE-045 trial, a randomized phase 3 trial that compared efficacy of pembrolizumab (200 mg q3w for up to 2 years) to chemotherapy (docetaxel, paclitaxel or vinflunine) in 524 patients with mUC who progressed during or after a platinum-based chemotherapy. After a median follow-up duration of 14.1 months, OS in all patients was significantly improved with pembrolizumab compared to chemotherapy (10.3 vs 7.4 months, respectively; HR 0.73, 95% CI 0.59 −0.91; *P* = 0.002). The median PFS was not significantly different between pembrolizumab and chemotherapy in all patients (2.1 vs 3.3 months, respectively; HR 0.98, 95% CI 0.81 to 1.19; *P* = 0.42).). The 12-month PFS rate was 16.8% in the pembrolizumab group and 6.2% in the chemotherapy group. The ORR was significantly better with pembrolizumab than with chemotherapy (21.1% versus 11.4%). Responses were durable with, at the time of data analysis, 18.4% of patients still receiving pembrolizumab compared to only 1.2% receiving chemotherapy. Interestingly, CPS was not associated with a better OS, PFS or ORR in pembrolizumab arm (see Table 1 for PD-L1 positivity). In the pembrolizumab arm, high PD-L1 expression was even associated with a lower OS compared to that of all pembrolizumab-treated patients (8 vs 10.1 months, respectively). In clinical practice, in second-line setting, PD-L1 status, based on CPS, is not helpful in selecting which patient would receive or not pembrolizumab [37].

Recent update of this trial showed that, after a median time from randomization to data cutoff of 6more than 5 years, pembrolizumab remains more active than chemotherapy in term of OS in overall population (10.1 vs 7.2 months, respectively; HR 0.71, CI 95% 0.59–0.86) and in patients with CPS ≥10 (8.0 vs 4.9 months, respectively; HR 0.59 (0.40–0.86). The 60-month OS rate was 14.9% and 8.7% in the pembrolizumab and in the chemotherapy group, respectively. OS benefit with pembrolizumab vs chemotherapy continued in all subgroups of patients. Median DOR for responders was longer for pembrolizumab vs chemotherapy (29.7 vs 4.4 months, respectively) [38]. Pembrolizumab represents thus a level-1 evidence in second-line setting, after failure of platinum-based therapy in mUC [38].

After promising resutls in a phase II single arm trial, atezolizumab was evaluated in a randomized phase III trial in second line setting, after failure of platinum-based chemotherapy [39]. The phase III IMvigor211 trial compared atezolizumab to standard second-line chemotherapy (vinflunine, paclitaxel, docetaxel) in 931 mUC patients after failure of platinum-based chemotherapy. The primary efficacy endpoint OS was to be tested in a hierarchical approach in study populations defined by IC PD-L1 expression, starting with high (IC 2/3) PD-L1 expression, followed by those with any level of PD-L1 expression (IC1/2/3), and followed by the ITT population. Statistical significance needed to be achieved in the IC2/3 population in order to evaluate the ITT population for statistical significance. Atezolizumab failed to demonstrated improved median OS compared to chemotherapy in IC2/3 PD-L1 expression patients (11.1 vs 10.6 months; HR 0.87, *P* = 0.41); there was no difference in the 1-year OS rate (46% vs 41%, respectively). Atezolizumab improved modestly, but significantly, the OS compared to chemotherapy in ITT population (8.6 vs 8.0 months; HR 0.85, *P* = 0.038). These perplexing results could be explained by the fact that the OS in the chemotherapy arm, and particularly in the vinflunine arm, appeared better than study design assumptions. One hypothesis is that the PD-L1 positive cohort was a smaller sample size and insufficiently powered to address the benefit in median OS for this cohort. The use of archival specimens may have confounded the true assessment of PD-L1-expression at the time of study entry. PD-L1 expression could also appear as a prognostic factor and selection of PD-L1 positive patients could have potentially selected patients with better prognosis, explaining a such impressive survival both in atezolizumab and in chemotherapy arm. Consistently with Imvigor210 results, the median DOR with atezolizumab was 21.7 months in the overall study population compared to 7.4 months with chemotherapy, confirming robust antitumoral efficacy of atezolizumab [38]. In light of these results and the better safety profile compared to chemotherapy, atezolizumab appears as an alternative to chemotherapy in second line metastatic setting in mUC, although the absence of level 1 [40].

Despite the absence of randomized trial, in February 2017, FDA approved nivolumab as second-line therapy based on large phase II single-arm CheckMate 275 study that enrolled 265 previously treated mUC patients. Nivolumab (3 mg/kg IV q2w) resulted in an ORR of 19.6% for the total population. The response rate was related to tumor PD-L1 expression; 28.4% for patients with tumor cell PD-L1 expression ≥ 5%, 23.8% for patients with tumor PD-L1 expression ≥ 1% and 16.1% for patients with low PD-L1 expression (< 1%). Median DOR was not reached and 77% of responses were ongoing at the time of analysis. The median OS was 8.74 months in all patients and increased to 11.3 months in PD-L1 ≥1% patients compared to 5.95 months in PD-L1 <1% patients [41].

Other ICIs, such as durvalumab and avelumab were evaluated in 2 phase 1/2 trials and showed efficacy in patients after failure of platinum-based therapy. The ORR reached up to 17.8% in overall population, but increased to 27.6% with durvalumab in PD-L1 high patients (defined as positive staining in ≥ 25% of tumor or immune cells). The median PFS was around 1.5 months in the entire population in both trials and OS reached 18.2 months in the entire population with durvalumab. All subgroups were beneficial [[42], [43], [44], [45]].

### Quest for optimal biomarker

ICIs play a key role in improving the outcome of patients with mUC. ICI is now the standard treatment in first-line setting with the maintenance strategy and in second-line setting after failure of platinum-based therapy, in patients progressing rapidly after perioperative chemotherapy. However, a proportion only of patients are responding to ICIs and ultimately, all patients present progressive disease. New emerging treatments are now evaluated after failure of ICIs, including antibody-drug conjugates (ADC) such as enfortumab vedotin (EV) or sacituzumab [46,47]. These ADC improve significantly the prognosis of mUC patients and large randomized phase III trials should precise the place of these agents in the management of patients. When considering randomized trials, control arm should include the maintenance strategy as this is, to date, the optimal regimen in first-line metastatic setting, with a significant 6-month survival benefit compared to historical regimen.

### Role of PD-L1 biomarker, not an ideal biomarker

Some of the different clinical trials evaluating ICIs in UC showed that PD-L1 expression could be associated with efficacy, while other did not. Even if we may expect a higher amplitude of benefit in PD-L1 positive patients, a proportion of PD-L1 negative patients may also benefit from ICIs. This could be explained by the fact that PD-L1 is not the ideal biomarker; PD-L1 expression is dynamic and heterogeneous between primary tumor and metastases, and could change with time and with prior therapies such as radiotherapy and chemotherapy. Furthermore, detection and interpretation assays of PD-L1 staining are not standardized, particularly regarding the cut-off and the kind of cells expressing PD-L1. Some studies measured PD-L1 in the tumor, some measure PD-L1 in immune-infiltrating cells, and some measure both, all with different PD-L1 antibodies (SP142, 22C3, 28–8, and 5H1). The assays use different cut-off for positivity, including 1%, 5%, and an IHC score based on a sliding range. This lack of standardized PD-L1 testing is an important limitation in the validation of PD-L1 as a predictive biomarker across trials. However, PD-L1 alone could not be sufficient as a valuable predictive tool [48].

### Tumor mutation burden and tumor gene expression

Other more promising biomarkers include tumor mutational burden (TMB), TILs, and gene expression profiles. TMB is relatively high in UC compared with other cancers, with a median of 7.2 versus 3.6 mutations per megabase, respectively. In the IMvigor 211 trial, exploratory biomarker analyses showed that higher TMB predicted OS only in favor of atezolizumab but not in favor of chemotherapy plus atezolizumab. These analyses showed also that PD-L1 expression positively correlated with tumor gene expression (TGE) but not with TMB. High PD-L1 and high TGE were associated with improved outcomes with both chemotherapy and atezolizumab [49].

The phase 2 NCT02553642 is currently ongoing to evaluate the relationship between TMB and response to nivolumab/ipilimumab in advanced bladder cancer.

Predictive biomarker analysis was also performed in CheckMate275 trial that evaluated nivolumab in mUC. Of 270 treated patients, 139 had evaluable TMB. Higher TMB was associated with improved ORR (HR 2.13, 95% CI 1.26–3.60; *P* < 0.05), PFS (HR 0.75, 95% CI 0.61–0.92), and OS (HR 0.73, 95% CI 0.58–0.91). The combination of TMB and PD-L1 status was a better predictor, compared to PD-L1 alone, of PFS (*P* = 0.0056) and OS (*P* = 0.013) [50].

A gene signature-based nomogram has been evaluated and was shown to be associated with ICI response, predicting OS in mUC patients treated with ICI therapy. Ten prognostic genes have been identified, including six OS-favorable genes and four OS-detrimental genes. Amongst them, *CDH18, CXCL10, FOXN4, SLC6A4, CXCL9,* and *PCDH11X* are highly associated with ICI response in patients with mUC, because of igh rates of somatic mutations and anomalies in DNA-damage repair genes [51].

### Molecular classification and ICIs

Based on the Tumor Cancer Genome Atlas (TCGA), 4 molecular UC cluster subsets have been identified, according to different genetic signature and outcome (luminal cluster I and II, basal cluster III and IV) [52]. The association between this classification and ICI sensitivity was evaluated in the IMvigor211 trial. Luminal cluster II was characterized by transcriptional signatures of high activated T effector cells density and high IC PD-L1 expression; this cluster is associated with high response to atezolizumab.

Luminal cluster I was associated with low density of CD8+ effector genes, low PD-L1 IC/TC expression; this cluster is associated with low response to atezolizumab.

Basal clusters III and IV were associated with high PD-L1 IC and TC expression, as well as CD8+ effector genes; a reduced ORR was observed in basal subtypes compared to luminal cluster II subtype, suggesting that other immunosuppressive factors exist in the basal subtypes [53]. TCGA subtypes classification should be thus prospectively evaluated in further clinical trials.

### Future therapeutic strategies

ICIs appears a good candidate for combination with other therapeutic agents. A new role could emerge for anti-angiogenic agents in the era of ICIs, as it is now well demonstrated that angiogenic factors can play a direct and indirect role in immunosuppressive tumor microenvironment. For example, VEGF inhibits the differentiation of progenitor cells into CD8+ and CD4+ T cells; furthermore, VEGF directly upregulates PD-L1 expression on CD8+ T cells, decreasing their cytotoxic function. There is thus a rational to hypothesize that anti-angiogenic therapy could enhance efficacy of ICIs, as observed in renal cancer. In a phase II trial, cabozantinib is evaluated with pembrolizumab in first-line for cisplatin-ineligible patients in the PemCab trial (NCT03534804) or with nivolumab ± ipilimumab in genitourinary malignancies including UC (NCT02496208) [54].

Association of ADC and ICIs could also appear as promising. EV is a monoclonal antibody targeting Nectin-4, a protein overexpressed in UC, and is combined with a potent cytotoxic microtubule inhibitor, monomethyl auristatin E (MMAE); the release of MMAE disrupts the microtubule network within the cell, inducing cell cycle arrest and apoptotic cell death. Pembrolizumab was associated with EV in the phase 1b dose escalation and expansion study (NCT03288545) in mUC patients. Forty-five mUC patients deemed cisplatin-ineligible received EV (1.25 mg/kg days 1 and 8 of every 3-week cycle) plus pembrolizumab (200 mg every 3 weeks). The ORR reached 73.3% including 15.6% CR and 22% had SD, resulting in a disease control rate of 93.3%. These results need, of course, further confirmation [55].

In a review of 81 MIUC specimens, somatic *BRCA 1/2, PALB2, FANCD2, ERCC2, ATM* genes mutations were detected in 3.7–12.3% [58]. The presence of DNA repair gene aberrations is associated with an increase in tumor mutation load and infiltration of lymphocytes in the tumor microenvironment. There is thus a rationale to evaluate the combination of PARP inhibitors with PD1/PDL1 inhibitors in UC [56].

Last, preclinical evidence showed a rationale for the combination of radiation with ICI. Radiotherapy can promote adaptive resistance through upregulation of PD-L1 on tumor cells, Association with radiation may be particularly valuable in the treatment of immunologically “cold” tumors, which are characterized by low levels of T cell infiltrate, low mutation burden and low sensitivity to ICIs. Radiation could enhance increased depth and duration of response by allowing infiltration of lymphocytes into the tumor and modulating tumor microenvironment [57]..

## Conclusion

Maintenance strategy appears as a new concept in the therapeutic management of mUC; introducing ICI directly after platinum-based therapy, without waiting for progression disease, increases the survival of patient compared to historical sequential treatment. In this strategy, cisplatin and carboplatin appear as two accepted options; particularly, cisplatin-ineligible patients should be treated in first-line metastatic setting with carboplatin-based regimen directly followed, in case of non-progressive disease, by avelumab (Fig. 2). This strategy should thus be incorporate when considering randomized-controlled studies. Further trials are ongoing in order to identify optimal biomarker.

Malignant cells develop different mechanisms to evade immune recognition, including the upregulation of immune checkpoints on tumor and tumor-specific lymphocytes, resulting in inhibition of activated T-cells. The most commonly investigated immune checkpoints are cytotoxic T lymphocyte-associated protein 4 (CTLA-4), programmed cell death protein-1 (PD-1) and programmed death ligand-1 (PD-L1). In the priming phase, CTLA-4 expressed on T-cell exerts its inhibitory effect by competing with CD28 and by binding to B7, resulting in T-cell inactivation in lymphoid tissues. In the effector phase, PD-1 is an inhibitory receptor expressed on T-cells. PD1 can suppress T cell activation by affecting T cell proliferation.

## CRediT authorship contribution statement

**Hélène Houssiau:** Conceptualization, Methodology, Data curation, Writing – original draft, Visualization, Investigation, Writing – review & editing. **Emmanuel Seront:** Conceptualization, Methodology, Data curation, Writing – original draft, Visualization, Investigation, Writing – review & editing, Supervision.

## Declaration of Competing Interest

None.
